# Correlation of Optic Nerve Microcirculation with Papillomacular Bundle Structure in Treatment Naive Normal Tension Glaucoma

**DOI:** 10.1155/2014/468908

**Published:** 2014-12-09

**Authors:** Wataru Kobayashi, Hiroshi Kunikata, Kazuko Omodaka, Kyousuke Togashi, Morin Ryu, Masahiro Akiba, Gaku Takeuchi, Tetsuya Yuasa, Toru Nakazawa

**Affiliations:** ^1^Department of Ophthalmology, Tohoku University Graduate School of Medicine, 1-1 Seiryo-machi, Aoba-ku, Sendai 980-8574, Japan; ^2^Department of Retinal Disease Control, Ophthalmology, Tohoku University Graduate School of Medicine, 1-1 Seiryo-machi, Aoba-ku, Sendai 980-8574, Japan; ^3^Department of Bio-Systems Engineering, Faculty of Engineering, Graduate School of Science and Engineering, Yamagata University, 4-3-16 Jonan, Yonezawa-shi, Yamagata 992-8510, Japan; ^4^Topcon Corporation, 75-1 Hasunuma-cho, Itabashi-ku, Tokyo 174-8580, Japan; ^5^Department of Advanced Ophthalmic Medicine, Tohoku University Graduate School of Medicine, 1-1 Seiryo-machi, Aoba-ku, Sendai 980-8574, Japan

## Abstract

*Purpose.* To assess the association between optic nerve head (ONH) microcirculation, central papillomacular bundle (CPB) structure, and visual function in eyes with treatment naive normal tension glaucoma (NTG).* Methods*. This study included 40 eyes of 40 patients with NTG and 20 eyes of 20 normal patients. We used laser speckle flowgraphy (LSFG) to measure mean blur rate (MBR) in all eyes and calculated the ratio of MBR in the horizontal quadrants of tissue area ONH (temporal/nasal ratio of MBR in the tissue area: T/N MT). Clinical findings also included retinal nerve fiber layer thickness (RNFLT) and ganglion cell complex thickness (GCCT) in the CPB and macular areas, best-corrected visual acuity (BCVA), mean deviation (MD), and refractive error.* Results*. T/N MT was correlated with both BCVA and MD. The OCT parameters most highly correlated with T/N MT were macular RNFLT and mid-CPB RNFLT. Furthermore, T/N MT, mid-CPB RNFLT, and macular RNFLT were higher in NTG than in normal eyes. A discrimination analysis revealed that T/N MT and refractive error were independent factors indicating NTG.* Conclusions*. Our results suggest that T/N MT is a candidate biomarker of NTG. Furthermore, T/N MT reflects visual function, including acuity and sensitivity, and CPB structure.

## 1. Introduction

Glaucoma affects over 70 million people and is the second most common cause of blindness worldwide [1, 2]. Normal tension glaucoma (NTG), despite being the most common type of glaucoma in Asia, is less well understood than primary open angle glaucoma (POAG) [3–5]. Previous reports have shown that there is a general relationship between optic nerve microcirculation and glaucoma [6–9]. This relationship, in particular the effect of decreased microcirculation in the optic nerve head (ONH) in glaucoma, is one of the most promising potential sources of new insights into the pathogenesis of NTG, along with such factors as oxidative stress and axonal flow impairment. Quick, easy, and noninvasive ways to measure ONH microcirculation would therefore be very helpful for the investigation of the role of reduced ONH blood flow in glaucoma.

Laser speckle flowgraphy (LSFG) is a promising method of measuring ONH microcirculation that relies on the laser speckle phenomenon. Recent innovations in LSFG have allowed the* in vivo* quantification of microcirculation in the ONH, choroid, and retinal vessels separately [10, 11]. The main measurement parameter of LSFG is mean blur rate (MBR) [12, 13], a quantitative index of retinal blood cell (RBC) velocity. MBR is currently used mainly to monitor changes over time in ONH or choroidal microcirculation in the same eye at the same site [11], because the laser speckle signal can be influenced not only by RBC velocity, but also by laser reflectance and absorption in the retinal tissue [10]. Thus, previous interindividual comparisons of LSFG data have relied on analyses of MBR waveforms or MBR ratios rather than direct analysis of quantitative MBR values [14, 15].

Changes in the retinal structure, including macular and circumpapillary retinal nerve fiber layer thickness (mRNFLT and cpRNFLT, resp.), have also been reported to be closely associated with NTG [16–18]. Previously, we demonstrated that temporal cpRNFLT is significantly correlated to visual acuity in patients with glaucoma [19]. This prompted the current investigation, in which we compared the ratio of MBR data for the temporal and nasal optic nerve, measured with LSFG, and the thickness of the RNFL and GCC in the macula and the central papillomacular bundle (CPB), using a newly developed OCT analysis program. We also investigated differences in these values in NTG patients and normal subjects. Additionally, our analysis included clinical findings such as visual acuity and standard automated perimetry (SAP) measurements of mean deviation (MD). The purpose of this study was thus to evaluate the relationship between optic nerve microcirculation and retinal structure/function in eyes with treatment naive NTG.

## 2. Subjects and Methods

### 2.1. Inclusion Criteria

This retrospective, cross-sectional study comprised 40 eyes of 40 Japanese adult patients with NTG. Data from 20 eyes of 20 normal subjects (>40 years old) were used for comparison. All the NTG patients exhibited glaucomatous optic neuropathy. The inclusion criteria were (1) treatment naive NTG, (2) age > 40 years old, (3) a spherical equivalent refractive error of >−7.00 diopters, and (4) a glaucomatous visual field meeting the Anderson-Patella classification. The exclusion criteria were (1) decimal visual acuity < 0.1, (2) cataracts with severity greater than grade 2 of the Emery-Little classification, and (3) the presence of macular diseases such as macular edema, macular degeneration, or epiretinal membrane. The baseline clinical parameters recorded for each patient were age, sex, and refractive error. The baseline best-corrected visual acuity (BCVA) was measured with a standard Japanese decimal visual acuity chart and converted to logarithm of the minimum angle of resolution (logMAR) for statistical analysis. IOP was measured with Goldmann applanation tonometry during the initial diagnosis of NTG, before any glaucoma medications were used by the patient. The study adhered to the tenets of the Declaration of Helsinki, and the protocols were approved by the Clinical Research Ethics Committee of Tohoku University Graduate School of Medicine.

### 2.2. Visual Field Analysis

MD was measured with the 30-2 program of the Humphrey field analyzer (HFA; Carl Zeiss Meditec, Dublin, CA, USA), using the Swedish interactive threshold algorithm (SITA) standard strategy. HFA examinations were performed within three months of the OCT measurements. Only reliable MD values were used, excluding examinations with <20% fixation errors and <33% false-positives or false-negatives.

### 2.3. Laser Speckle Flowgraphy

ONH microcirculation was evaluated by measuring MBR in the optic disc with LSFG-NAVI (Softcare Ltd., Fukuoka, Japan). Ophthalmic examinations including slit-lamp biomicroscopy and gonioscopy were performed, and patients with narrow angles were excluded. LSFG measurements were carried out after dilation of the pupil with 0.4% tropicamide (Midrin-M, Santen Pharmaceutical Co. Ltd., Osaka, Japan). Before the LSFG examination, the patients rested on a chair with their eyes closed for 10 minutes in a dark room and measured their blood pressure. All examinations were performed by experienced investigators. Edge detection of the optic disc in the MBR image was performed manually and the disc edge was saved in software. The vessels were then segmented in the supplied software (LSFG Analyzer, v 3.0.47.0) with an automated defining threshold, and the values of mean MBR (MA), MBR in the vessel area (MV), and MBR in tissue area (MT) were determined. These values were determined separately for each quadrant of the ONH: superior (S), inferior (I), temporal (T), and nasal area (N), as well as overall. Triplicate measurements were made of each subject, separated by several minutes, using the saved data for the optic disc edge.

### 2.4. OCT Scanning of the Disc and Macular Areas

CpRNFLT, mRNFLT, and GCCT were determined with 3D OCT-2000 software (version 8.00; Topcon Inc.). After obtaining circle scans and macular cube scans (in a 7 × 7 mm area corresponding to 10-degree square area of the macula) centered on the fovea, the software automatically calculated the thickness of each layer.

### 2.5. OCT Scanning of the Central Papillomacular Bundle

The CPB was defined in this study as a 1.5 × 9.0 mm rectangular area centered between the optic nerve disc and the macula, aligned perpendicularly to the nerve fibers. At either end of the scan area, a 1.5 × 1.2 mm area in which the retinal layers could not be reliably segmented was discarded. The remaining 1.5 × 6.6 mm area was divided lengthwise into three 1.5 × 2.2 mm sections, representing the upper, middle, and lower CPB. Analysis of the CPB used 3D OCT images, obtained with the 3D OCT-2000 (Topcon Corporation, Tokyo, Japan) device. Each image was constructed from 64 B-scan images (pixel dimensions: 512 × 885, grayscale levels: 256) with depth and lateral resolutions of 6 *μ*m and 20 *μ*m, respectively. Layer segmentation was performed with newly developed software (Topcon). The RNFLT and GCCT of the CPB were measured with automatic analysis software developed by the Graduate School of Science and Engineering, Yamagata University. This software was equipped with a registration system (using a fast registration algorithm for the 3D OCT images based on en-face projection images) to ensure the reliability and reproducibility of the clinical data.

### 2.6. Statistical Analysis

Spearman's correlation analysis was used to determine the correlation between the data from the structural examinations, that is, RNFLT and GCCT, and the data from the functional tests, that is, MD and BCVA (logMAR), as well as the ratio of tissue area MBR in the horizontal quadrants, that is, the temporal and nasal tissue area MBR (T/N MT). The Mann-Whitney *U* test was used to determine differences between the NTG patients and normal subjects. A discrimination analysis was used to determine the predictive power of the measurement parameters for NTG. The analysis used JMP Pro version 9.0.2 software for Windows (SAS Institute, Japan). A *P* value < 0.05 was considered to be statistically significant.

## 3. Results

**Figure 1:**
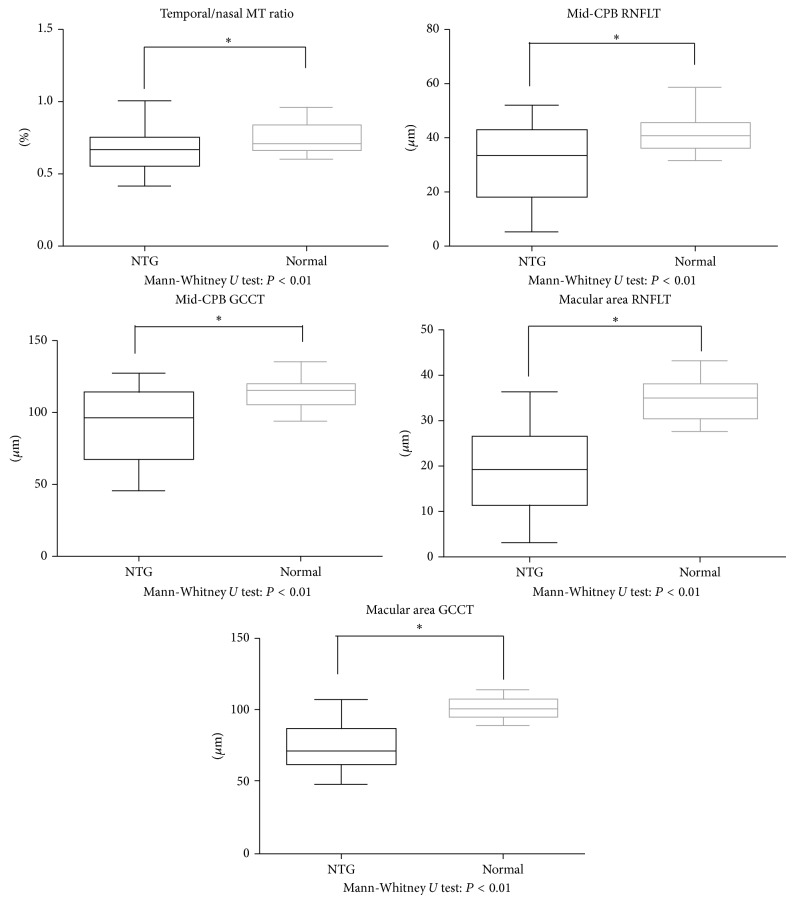
Comparison of T/N MT, RNFLT, and GCCT in NTG patients and normal subjects. T/N MT, mid-CPB RNFLT, mid-CPB GCCT, macular RNFLT, and macular GCCT were decreased in NTG patients compared to normal subjects (all *P* < 0.01).

**Figure 2:**
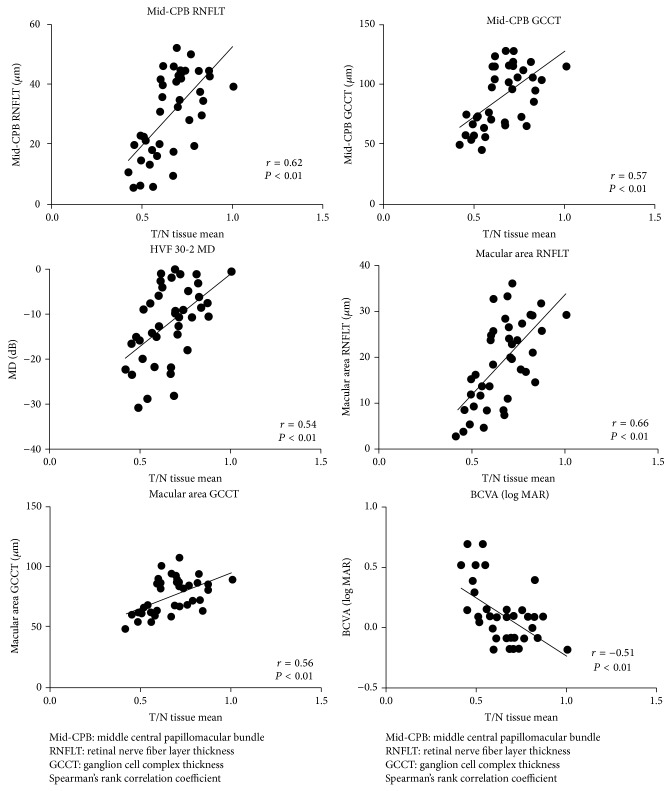
Correlation of T/N MT with MD, BCVA, RNFLT, and GCCT. In NTG patients, T/N MT was correlated with mid-CPB RNFLT, mid-CPB GCCT, macular RNFLT, macular GCCT, MD, and BCVA (all *P* < 0.01).

Table 1 shows demographic information and biometric parameters for the NTG patients and normal subjects.  Table 2 shows the correlations of these parameters with T MT, I MT, N MT, S MT, and T/N MT. T MT was most closely correlated with all but one of the OCT parameters. T/N MT was also closely correlated with all parameters. The parameters most strongly correlated with T/N MT were mid-CPB RNFLT, mRNFLT, mid-CPB GCCT, and macular GCCT (*r* = 0.62, *r* = 0.66, *r* = 0.57, and *r* = 0.56, resp., all *P* < 0.01).  Figure 1 shows comparisons of T/N MT and retinal thickness (in the mid-CPB and macular areas) in NTG patients and normal subjects. We found significant differences between the NTG patients and normal subjects in T/N MT and mid-CPB and macular retinal thickness (all *P* < 0.01).  Figure 2 shows the correlation of T/N MT and retinal thickness (in the mid-CPB and macular areas) and MD and BCVA (logMAR) (all *P* < 0.01).

A discrimination analysis including 20 NTG patients and 20 age-matched normal subjects revealed that T/N MT and refractive error were independent factors able to distinguish NTG patients from normal subjects (*P* < 0.05, Table 3). The hit ratio of correctly classified cases was 80.0%.

## 4. Discussion

In this study, we examined the relationship between novel analytic parameters of temporal optic nerve microcirculation and papillomacular bundle thickness in NTG patients. Among these parameters, we found that T/N MT, the ratio of optic nerve microcirculation in the temporal quadrant, was strongly correlated with RNFLT in the mid-CPB and macular areas and with GCCT in the mid-CPB and macular areas. We also found correlations between T/N MT and total mid-CPB and macular thickness as well as correlations between T/N MT and visual function, including visual acuity and MD.

It is well known that, in glaucoma, the eye undergoes loss of the retinal GCL and of the axons that comprise the RNFL [20]. The pathogenesis of these phenomena remains unclear, but changes in ocular blood flow, especially decreases in ONH microcirculation, have recently been reported to play an important role, particularly in the occurrence and progression of NTG [21, 22]. This new understanding of the role of ocular blood flow may help explain why lowering IOP treatment, despite being an effective, gold-standard treatment for POAG, has limitations when used for NTG [23]. Epidemiological studies including the Singapore Malays Eyes Study [24] and the Beijing Eye Study [25] have shown that the prevalence of glaucoma increases with retinal arteriole or vessel narrowing, and the Blue Mountain Eye Study showed that glaucoma was 2.7 times more likely in eyes with retinal arteriole narrowing [26]. A review of recent research on glaucoma revealed a number of reports on the role of dysfunctional ocular blood flow in the prevalence and progression of glaucoma [27–29], reinforcing our findings. Previous findings in which the highest correlation between visual acuity and RNFLT was in the temporal sector prompted our study's focus on temporal ONH microcirculation [19]. As it is well known that visual acuity decreases only in the end stages of glaucoma, it has been thought that the macula in eyes with glaucoma only rarely becomes disordered [30]. In fact, recent reports have shown that damage to the macula can occur even in the early stages of glaucoma [31, 32]. Our study may help explain these previous findings, as our results suggest that decreases in temporal optic nerve blood flow may affect the structure of the CPB and macula, leading to decreases in macular function, including visual acuity.

Laser speckle flowgraphy (LSFG) is a new and increasingly commonly used technology that can quantify microcirculation in the optic disc, choroid, and retinal vessels separately. It is noninvasive, accurate, and flexible and is therefore suitable for studying many aspects of ocular circulation [11]. MBR, the main measurement parameter of LSFG, is a relative measurement index of the velocity of erythrocytes [12, 13]. ONH filling defect area, measured with fluorescein angiography (FA), is also used in studies of ocular blood flow and is correlated to glaucomatous damage [33–37]. It is difficult to include FA as a part of clinical care for glaucoma, however, as quantitative measurements of changes in ONH blood flow cannot be obtained with sufficient reliability. Other methods to quantify blood flow have also been reported, such as scanning laser ophthalmoscopy-FA, which can measure decreases in fluorescein velocity in eyes with glaucoma [8], single-point laser Doppler flowmetry, which can identify decreased ONH blood flow in glaucoma [6], and scanning laser Doppler flowmetry, which has been used to show that retinal blood flow in both the neuroretinal rim and peripapillary area is decreased in glaucoma [7]. Scanning laser Doppler flowmetry has also been used to show that perfusion in the ONH can decrease before the manifestation of visual field defects [38]. In contrast with these techniques, LSFG measurements of MBR can be obtained easily and quickly (within a few seconds) and are highly reproducible in both normal and glaucoma subjects [39]. In our study, we investigated the role of ocular microcirculation in glaucoma by determining the ratio of MBR in the temporal and nasal quadrants of the ONH tissue area. We found that, among the quadrants of the ONH, temporal MBR was most closely correlated with a number of parameters of retinal thickness, while nasal MBR was the least. We thus calculated the ratio between MBR in these two quadrants in order to obtain a value that was comparable between eyes. The correlation between temporal MBR and other measurement parameters reflects the close relationship between temporal ONH microcirculation and central visual function, while the lack of correlation of nasal MBR reflects the common preservation of nasal ONH microcirculation in glaucoma patients, despite the loss of visual function [14, 19].

Currently, the diagnosis of glaucoma is mainly made based on the presence of pronounced optic disc cupping, decreased cpRNFLT, and progressive visual field loss. Recently, spectral domain optical coherence tomography (SD-OCT) technology has been introduced, which enables the visualization of each retinal layer in the macular area, including the GCC, with programmed segmentation algorithms [40]. In glaucoma patients, the macular area is impaired because of changes in the RNFL and GCC of the inner retina [41]. Several studies have reported that macular GCCT and cpRNFLT have a very similar diagnostic potential for glaucoma [16, 17, 41]. The current study both confirmed the value of conventional measurements of macular thickness and found that a new measurement program for CPB thickness may also be helpful, as it was closely correlated with temporal ONH circulation. This new measurement parameter could identify NTG and was associated with visual acuity and sensitivity.

This study had a number of limitations. It included only a small number of eyes with glaucoma (approximately 40) and was retrospective. The study methodology introduced the potential for bias, as myopia in the patients could have affected our results for the correlation between structure and function. We therefore excluded NTG patients with high myopia (less than −7 diopters) and there was no statistical difference on refractive error between NTG and normal subjects. Nevertheless, refractive error was present in our results as a weak independent factor differentiating NTG. Furthermore, although there were significant differences in the T/N MT ratio between normal and NTG eyes, the data for the two groups overlapped a fair amount. Nevertheless, when the analysis also includes simple clinical parameters such as age, IOP, and refractive error, its hit rate for detecting NTG is 80%, sufficient for it to be considered a good clinical indicator.

In this study, we examined the relationship between structure/function and microcirculation in eyes with NTG. We found that the ratio of temporal ONH blood flow was strongly correlated with mid-CPB and macular thickness as well as BCVA and MD, and that measurement of temporal ONH microcirculation may be an excellent way to determine whether thinning of the RNFL and GCC in glaucoma patients is due to NTG.

## Figures and Tables

**Table 1:** Characteristics of patients.

Characteristics	NTG	Normal people	*P* value
	Number, mean ± SD	Number, mean ± SD	
Number	40	20	—
Gender			
Male	19 (19 eyes)	10 (10 eyes)	0.81^*^
Female	21 (21 eyes)	10 (10 eyes)	
Laterality			
Right	21	9	0.81^*^
Left	19	11	
Age (years)	63.3 ± 9.6	59.7 ± 9.9	0.16^**^
BCVA (log⁡MAR)	0.10 ± 0.2	−0.10 ± 0.08	<0.01^**^
IOP (mmHg)	14.6 ± 3.2	16.2 ± 3.3	0.18^**^
Refractive error (diopter)	−2.94 ± 2.3	−2.04 ± 2.2	0.17^**^
HFA 30–2 mean deviation (MD; dB)	−11.9 ± 8.4	0.17 ± 1.3	<0.01^**^
Macular area			
RNFLT (*μ*m)	19.1 ± 9.1	34.5 ± 4.0	<0.01^**^
GCCT (*μ*m)	75.3 ± 14.7	101.6 ± 7.0	<0.01^**^
Central papillomacular bundle			
RNFLT (*μ*m)			
Upper	36.4 ± 21.2	66.9 ± 15.6	<0.01^**^
Middle	30.3 ± 13.9	42.2 ± 7.7	<0.01^**^
Lower	29.1 ± 21.0	70.9 ± 18.1	<0.01^**^
GCCT (*μ*m)			
Upper	81.5 ± 25.1	117.2 ± 14.2	<0.01^**^
Middle	90.7 ± 24.9	114.5 ± 9.5	<0.01^**^
Lower	75.3 ± 21.4	120.7 ± 17.7	<0.01^**^
Circumpapillary RNFLT (*μ*m)			
Total	81.2 ± 12.2	107.3 ± 10.6	<0.01^**^
Temporal	71.1 ± 17.5	84.9 ± 11.5	<0.01^**^
Superior	98.3 ± 19.2	126.6 ± 16.3	<0.01^**^
Nasal	75.1 ± 16.3	86.1 ± 15.9	<0.01^**^
Inferior	80.4 ± 20.6	131.5 ± 17.0	<0.01^**^
LSFG MBR (AU)			
MA			
Temporal	8.6 ± 2.6	11.8 ± 2.1	—
Superior	20.4 ± 6.1	28.3 ± 5.6	—
Nasal	21.5 ± 6.3	29.9 ± 6.4	—
Inferior	17.5 ± 5.0	26.7 ± 5.3	—
MT			
Temporal	7.8 ± 2.0	10.1 ± 1.6	—
Superior	10.8 ± 2.7	12.6 ± 2.0	—
Nasal	11.9 ± 2.5	13.5 ± 1.6	—
Inferior	10.0 ± 2.4	12.9 ± 1.8	—
MV			
Temporal	27.2 ± 10.1	36.9 ± 12.7	—
Superior	37.9 ± 9.0	48.0 ± 5.2	—
Nasal	36.3 ± 8.9	48.4 ± 7.6	—
Inferior	35.1 ± 7.7	44.7 ± 5.1	—
Temporal/nasal MT	0.66 ± 0.1	0.75 ± 0.1	<0.01^**^
Systolic blood pressure (SBP) (mmHg)	127.1 ± 20.4	133.7 ± 21.7	0.26^**^
Diastolic blood pressure (DBP) (mmHg)	77.8 ± 14.5	82.9 ± 14.1	0.07^**^
Heart rate (HR) (bpm)	73.2 ± 11.1	76.9 ± 9.9	0.16^**^

NTG: normal tension glaucoma, BCVA: best-corrected visual acuity, log⁡MAR: logarithm of the minimal angle resolution, IOP: intraocular pressure, HFA: Humphrey field analyzer, RNFLT: retinal nerve fiber layer thickness, GCCT: ganglion cell complex thickness, MBR: mean blur rate, AU: arbitrary unit, MA: mean MBR in all areas, MT: mean MBR in the tissue area, and MV: mean MBR in the vessel area.

^*^Fisher's exact test, ^**^Mann-Whitney *U* test.

**Table 2:** Correlation of retinal thickness with optic disc microcirculation in NTG.

	Age	IOP	Ref.	BCVA (log⁡MAR)	MD	Rectangular		Rectangular		Rectangular		Central part of the macula		cpRNFLT	
						Upper1/3		Middle		Lower1/3					
						RNFLT	GCCT	RNFLT	GCCT	RNFLT	GCCT	RNFLT	GCCT	Total	Temporal
T MT	−0.07	−0.05	−0.04	−0.49^**^	0.57^**^	0.49^**^	0.45^**^	0.66^**^	0.68^**^	0.32^*^	0.30^*^	0.59^**^	0.63^**^	0.45^**^	0.48^**^
I MT	−0.12	−0.05	−0.08	−0.29	0.32^*^	0.33^*^	0.41^**^	0.47^**^	0.54^**^	0.24	0.31^*^	0.44^**^	0.50^**^	0.45^**^	0.40^**^
N MT	−0.04	−0.23	0.19	−0.05	0.19	0.09	0.18	0.12	0.22	0.03	−0.01	0.09	0.18	0.37^*^	0.22
S MT	−0.12	−0.12	0.03	−0.38^*^	0.43^**^	0.47^**^	0.62^**^	0.44^**^	0.57^**^	0.01	0.11	0.42^**^	0.53^**^	0.42^**^	0.33^*^
T/N MT	−0.09	0.15	−0.29	−0.51^**^	0.54^**^	0.54^**^	0.39^*^	0.62^**^	0.57^**^	0.47^**^	0.39^*^	0.66^**^	0.56^**^	0.25	0.36^*^

IOP: intraocular pressure, Ref.: refractive error, BCVA: best-corrected visual acuity, log⁡MAR: logarithm of the minimal angle resolution, MD: HFA 30–2 mean deviation, RNFLT: retinal nerve fiber layer thickness, GCCT: ganglion cell complex thickness, cpRNFLT: circumpapillary retinal nerve fiber layer thickness, MT: mean MBR in the tissue area, T: temporal, I: inferior, N: nasal, and S: superior.

Spearman's rank correlation coefficient, ^*^ *P* < 0.05, and ^**^ *P* < 0.01.

**(a):** 

Variable	Wilks's lambda	*F*-number	*P* value
Age	0.9560	1.6104	0.32
IOP	0.8967	4.0329	0.06
Ref.	0.8383	6.7496	<0.05
T/N MT	0.6478	19.0278	<0.01

**(b):** 

Judgment result	Predicted value		
	Normal	NTG	Hit ratio
Observed value			
Normal	16	4	80.0%
NTG	4	16	80.0%

		Total	80.0%
